# Supplementary material for: Methodological quality of cohort study on rheumatic diseases in China: A systematic review
Source: PLoS One. 2020 Apr 23;15(4):e0232020. doi: 10.1371/journal.pone.0232020 (PMC7179908; doi:10.1371/journal.pone.0232020)
Supplement: S1 Fig — (DOC) [file pone.0232020.s002.doc]

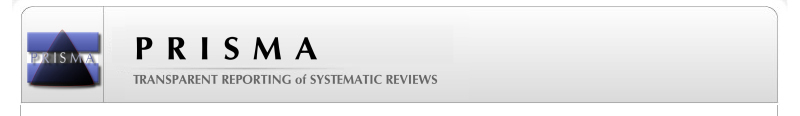
**PRISMA 2009 Flow Diagram**

**Screening**

**Included**

**Eligibility**

**Identification**

Records identified through database searching
(n = 623)

Additional records identified through other sources
(n = 0)

Records after duplicates removed
(n = 442)

Records screened
(n = 442)

Records excluded
(n = 350)

Full-text articles assessed for eligibility
(n = 92)

Full-text articles excluded, with non-standardized design
(n = 46)

Studies included in qualitative synthesis
(n = 46)

Studies included in quantitative synthesis (meta-analysis)
(n = 46)
