# Supplementary material for: Methodological quality of cohort study on rheumatic diseases in China: A systematic review
Source: PLoS One. 2020 Apr 23;15(4):e0232020. doi: 10.1371/journal.pone.0232020 (PMC7179908; doi:10.1371/journal.pone.0232020)
Supplement: Attachment — Submitted filename: Response to Reviewers.docx [file pone.0232020.s003.docx]

**Manuscript Title:** Methodological Quality of cohort study on rheumatic diseases in China” (PONE-D-19-24922) By Huan Zhang, Guoxiang Yi, Mingzhu Dai, Yanping Li and Bin Wu.

Followings are the detailed responses for the comments. The blue words are comments and the black are reply.

**Major comments**

**1. When submitting your revision, we need you to address these additional requirements.**

**Please ensure that your manuscript meets PLOS ONE's style requirements, including those for file naming. The PLOS ONE style templates can be found at**

[**http://www.journals.plos.org/plosone/s/file?id=wjVg/PLOSOne_formatting_sample_main_body.pdf**](http://www.journals.plos.org/plosone/s/file?id=wjVg/PLOSOne_formatting_sample_main_body.pdf) **and** [**http://www.journals.plos.org/ plosone/s/file?id=**](http://www.journals.plos.org/plosone/s/file?id=)**ba62/PLOSOne_formatting_sample_title_authors _affiliations.pdf**

**Response:** We have browsed the website and checked our manuscript to ensure it meets PLOS ONE's style requirements.

**2. During our internal evaluation of your manuscript, we noticed several typos. For instances, line 37: Englishstudies, line 38: selectionofstatistical, line 176:Table 2.t. Please note that PLOS ONE does not copyedit accepted manuscripts, so please ensure the language in submitted articles is clear, correct, and unambiguous.**

**Response:** Sorry, this is our carelessness.

**Emendations:** We have corrected them one by one.

line 27: the statement of “andanalyzed” was corrected as “and analyzed”;

line 34: the statement of “Englishstudies” was corrected as “English studies”;

line 35: the statement of “selectionofstatistical” was corrected as “selection of statistical”;

line 60: the statement of “pneumoniaunless” was corrected as “pneumonia unless”;

line 162: the statement of “Table 2.t” was corrected as “Table 2. It”;

line 234: the statement of “underrepresentation” was corrected as “under representation”.

line 240: the statement of “literature.However” was corrected as “literature. However”.

line 245: the statement of “developedlate” was corrected as “developed late”.

line 253: the statement of “points.This” was corrected as “points. This”.

line 254: the statement of “papersduring” was corrected as “papers during”.

**3. Please include captions for your Supporting Information files at the end of your manuscript, and update any in-text citations to match accordingly. Please see our Supporting Information guidelines for more information:** [**http://journals.plos.org/plosone/s/supporting**](http://journals.plos.org/plosone/s/supporting)**- information.**

**Response:** All data and information have been presented in the manuscript, without additional information to supplement.

4. **Please confirm that you have included all items recommended in the PRISMA checklist including identifying the study as a meta-analysis or systematic review in the title.**

**Response:** We confirmed that we have included all items recommended in the PRISMA checklist and add “a systematic review” in the title.

5. **The manuscript needs English editing by a native English speaker to improve the quality of language.**

**Response:** Ok! To improve English expression, we invited International Science Editing to polish and edit this paper.

**Emendations:** The corrections in the paper are as following:

We have rewritten this part according to the requirements of the magazine. The comment symbol for the author's name was changed and Current Address was added. In addition, the information format of corresponding author has been changed.

We have fixed the font format for headings at all levels.

We upload our figure files to the Preflight Analysis and Conversion Engine (PACE) digital diagnostic tool to ensure that figures meet PLOS requirements. Besides, the titles, legends, comment marks of the pictures and tables were changed as required.

All references are supplemented as much as possible with doi and PMID, and Article 59 references have been updated.

We corrected three sentences.

Line 46: the sentence “The quality of the research was generally similar, and all reports were insufficient, in both the English and Chinese literature.” was corrected as “The quality of Chinese studies was better than English studies, and all reports were insufficient.”

Line 213: the sentence “A retrospective cohort of 37,338 twins across Denmark showed that genes play only a minor role in the pathogenesis of rheumatoid arthritis” was corrected as “the Framingham cohort study in the United States on the risk factors for osteoarthritis found that meniscal injury in the middle-aged and elderly populations increased with age and had no parallel relationship with clinical symptoms.”

Line 291: the sentence “On the other hand, the results of the NOS quality evaluation suggest that the quality of the study is similar in Chinese and English reports.” was corrected as “On the other hand, NOS quality assessment results show that the quality of Chinese reports is better than English reports.”

Some changes that do not affect the meaning of the content are not listed, but they are marked in red in the article.
